# Body mass index and incident coronary heart disease in women: a population-based prospective study

**DOI:** 10.1186/1741-7015-11-87

**Published:** 2013-04-02

**Authors:** Dexter Canoy, Benjamin J Cairns, Angela Balkwill, F Lucy Wright, Jane Green, Gillian Reeves, Valerie Beral

**Affiliations:** 1 Cancer Epidemiology Unit, University of Oxford, Roosevelt Drive, Oxford, OX3 7LF, UK

**Keywords:** obesity, body mass index, coronary heart disease, incidence, mortality, women

## Abstract

**Background:**

A high body mass index (BMI) is associated with an increased risk of mortality from coronary heart disease (CHD); however, a low BMI may also be associated with an increased mortality risk. There is limited information on the relation of incident CHD risk across a wide range of BMI, particularly in women. We examined the relation between BMI and incident CHD overall and across different risk factors of the disease in the Million Women Study.

**Methods:**

1.2 million women (mean age = 56 years) participants without heart disease, stroke, or cancer (except non-melanoma skin cancer) at baseline (1996 to 2001) were followed prospectively for 9 years on average. Adjusted relative risks and 20-year cumulative incidence from age 55 to 74 years were calculated for CHD using Cox regression.

**Results:**

After excluding the first 4 years of follow-up, we found that 32,465 women had a first coronary event (hospitalization or death) during follow-up. The adjusted relative risk for incident CHD per 5 kg/m^2 ^increase in BMI was 1.23 (95% confidence interval (CI) 1.22 to 1.25). The cumulative incidence of CHD from age 55 to 74 years increased progressively with BMI, from 1 in 11 (95% CI 1 in10 to 12) for BMI of 20 kg/m^2^, to 1 in 6(95% CI 1 in 5 to 7) for BMI of 34 kg/m^2^. A 10 kg/m^2 ^increase in BMI conferred a similar risk to a 5-year increment in chronological age. The 20 year cumulative incidence increased with BMI in smokers and non-smokers, alcohol drinkers and non-drinkers, physically active and inactive, and in the upper and lower socioeconomic classes. In contrast to incident disease, the relation between BMI and CHD mortality (*n *= 2,431) was J-shaped. For the less than 20 kg/m^2 ^and ≥35 kg/m^2 ^BMI categories, the respective relative risks were 1.27 (95% CI 1.06 to 1.53) and 2.84 (95% CI 2.51 to 3.21) for CHD deaths, and 0.89 (95% CI 0.83 to 0.94) and 1.85 (95% CI 1.78 to 1.92) for incident CHD.

**Conclusions:**

CHD incidence in women increases progressively with BMI, an association consistently seen in different subgroups. The shape of the relation with BMI differs for incident and fatal disease.

## Background

There is growing concern about the impact of the increasing prevalence of obesity on the burden of coronary heart disease (CHD) [1], which accounts for around 15% of all deaths in the UK, USA, and other developed nations [2-4]. Reducing this obesity-associated CHD burden is likely to require population-level preventive strategies. One such approach suggests shifting the population distribution of a modifiable risk factor downwards to prevent the occurrence of CHD [5], but it is unclear if this approach is relevant for obesity. Most large-scale prospective studies have reported on CHD mortality outcomes, showing increased coronary mortality risks in both high and low BMI groups [6-8]. The relation between BMI and CHD may differ for incident and fatal disease [9-11], but such findings were based on a relatively small number of events. Large-scale studies that reported on incident CHD outcomes have been limited, particularly in women, and have not compared findings for incident and fatal CHD [12-17]. It is also unclear if the relation between BMI and incident CHD varies by age [8,15] or by lifestyle risk factors, particularly smoking [15,18,19]. Large-scale prospective studies may be needed to provide reliable risk estimates for incident CHD across a wide range of BMI in the whole population and across important subgroups. To address these questions, we examined the relation between BMI and CHD incidence and mortality in a cohort of over a million women followed for an average of 9 years.

## Methods

The Million Women Study is a population-based cohort study that recruited 1.3 million women who were invited for routine breast cancer screening between 1996 and 2001 in England and Scotland by the National Health Service (NHS) screening program [20]. At recruitment, women completed a health and lifestyle questionnaire, which included questions on weight, height, sociodemographic details, medical history, and lifestyle habits. All participants gave their written consent to take part in the study. The Oxford and Anglia Multi-Centre Research Ethics Committee approved the conduct of this study.

Using their individual NHS identification number, together with other personal information, participants were linked to NHS Central Registers for information on deaths, cancer registration, and emigrations, and to the NHS hospital admission databases for information on hospital admissions. For participants in England, the Hospital Episode Statistics data were available from 1 April 1997; for participants in Scotland, the Scottish Morbidity Records [21,22] data were available from 1 January 1981. Hospital diagnoses and causes of death were coded using the *International Statistical Classification of Diseases and Related Health Problems*, Tenth Revision [23] (ICD-10).

### Calculation, definition, and validation of anthropometric variables

We used BMI (weight (kg) divided by height (m^2^), and rounded to the nearest tenth) as our primary measure of adiposity, as it is strongly correlated with total fat mass [24] and provides a comparable explanatory power of the physiologic effect of total fat mass [25]. Overweight and obesity were defined as a BMI of 25 to 29.9 and ≥30 kg/m^2^, respectively. Self-reported weight and height were used to calculate BMI, and two different sources were used to validate the self-reported measures. We identified 541 women who were also participants in another longitudinal study and for whom their weight and height had been measured at the age of 53 years (at around the time that they reported their weight and height to us), and the correlation between BMI calculated from the measured and the self-reported data was 0.90 [26]. A sample of 3,745 women had their weight and height measured at general practice clinics in 2006 and 2008. BMI calculated from self-reported weight and height at baseline on average was 1.4 ± 2.5 kg/m^2 ^(mean ± SD) lower than that from the clinical measurements, taken some 8 to 10 years later, and the correlation between them was 0.85. In a sensitivity analysis, we used data from the clinical measurements to evaluate the effect of measurement error, including changes in BMI over time, on risk estimates [27].

### Definition and validation of outcomes

We defined an incident CHD event as the first hospital admission after recruitment with a diagnosis of CHD (ICD-10 I20 to I25) or death with CHD as the underlying cause. In a validation study, we randomly selected 796 women with a hospital record of CHD and 864 with no admission for vascular disease [28]. We asked general practitioners (GPs; they hold the medical record of every individual registered with the NHS) to report if these women had been given this diagnosis and to provide us with the relevant clinical information to support this report. An adjudicating team (FLW, DC, BJC, AB, and JG) reviewed the GPs' diagnosis and the clinical information provided to the investigators. The GP diagnosis of CHD was consistent in 92% of 796 women with a hospital record of CHD; in addition to 864 women without vascular disease hospitalization, 98% were confirmed to have had no CHD diagnosis.

### Analysis

Of the 1.3 million women recruited into the study, we excluded 78,895 (5.8%) women who reported heart disease or stroke at recruitment or had been admitted to hospital for these conditions before study entry, and 44,803 (3.3%) women who had a previous cancer registration (except non-melanoma skin cancer), as cancer may affect weight. Of the remaining 1.2 million women, we further excluded 64,620 (5%) women for whom BMI values were missing. The remaining 1,178,939 women formed the basis for our analysis.

We used Cox regression models to calculate hazard ratios to estimate relative risks of CHD separately for incident and mortality outcomes, using attained age as the underlying time variable. Person-years were calculated from date of recruitment until date of first admission for CHD, death, or end of follow-up, whichever came first. Around 5% of participants in England were recruited before 1 April 1997, and as hospital admission data before this date were not available, their follow-up was calculated from this date. The follow-up of women ended on 31 March 2008 in England and 31 December 2008 in Scotland, because hospital admission data were not complete after these dates. The regression models were stratified by region of recruitment (10 regions) and adjusted for smoking (never, past and current smokers with consumptions of less than 5, 5 to 9, 10 to 14, 15 to 19, 20 to 24 and ≥25 cigarettes per day), weekly alcohol consumption (0, 1 to 6, 7 to 14 and ≥15 U), strenuous physical activity (rarely/never, once a week or less, and more than once a week), and socioeconomic level (fifths of Townsend deprivation index [29]). There were a few missing values for smoking (0.7%), physical activity (3.0%), alcohol intake (0.6%) and socioeconomic status (0.7%). For each adjustment variable, women with missing values were assigned to a separate category.

We first calculated the incidence rate of CHD for each year of follow-up to examine variations in disease rates over time. Absolute risks of CHD were then calculated as cause-specific cumulative incidences. Within 5-year age groups (55 to 59, 60 to 64, 65 to 69, and 70 to 74 years), incidence rates were computed from the number of coronary events and time at risk. Hazard ratios from Cox regressions were converted to absolute hazard rates by multiplying the hazard ratios by the overall incidence rate, divided by a weighted average of the BMI category-specific hazard (weights were given by the total person-time at risk for women in the corresponding BMI category) [8]. Cause-specific cumulative incidence over 5 years was calculated for each age and BMI group, where appropriate, from the absolute hazard *h *(in units of events per person per year) by the formula $\text{1}-\text{exp}\left(-5h\right)$. The 20-year cause-specific cumulative incidence between the ages of 55 and 74 years was calculated from the simple average of the absolute hazards across the four age groups, $\bar{h}$, by the formula $\text{1}-\text{exp}\left(-\text{2}0\bar{h}\right)$. We estimated the 20-year cumulative incidence for the whole cohort and by subgroups of women classified by smoking habit, physical activity, alcohol consumption, and socioeconomic status.

We present the risk estimates with their 95% CI. When comparing more than two groups (such as in a figure), relative risks are presented with their 95% group-specific CI (g-sCI) to allow direct comparison between any two groups [30], even if neither is the baseline group. We conducted sensitivity analyses to assess the effects of missing values by comparing the relative risks based on data for all women with those women who had no missing values for any covariate, and to evaluate the effects of competing causes of death on the absolute risk estimates [31]. All analyses used Stata 12.0 (StataCorp., College Station, TX, USA) [32].

## Results

The characteristics of the 1,178,939 women included in the analyses are shown in Table 1. The mean age of women at recruitment was 56.0 ± 4.8 years (25^th ^to 75^th ^percentile range 52 to 60) and the mean BMI was 26.1 ± 4.6 kg/m^2^. The proportions of overweight and obese women at baseline were 35.3% and 17.1%, respectively, with 5.4% having a BMI of 35 kg/m^2 ^or over. Mean alcohol consumption of alcohol drinkers was 4.2 ± 5.4 U/week. Compared with women with a BMI of 20 to 24.9 kg/m^2^, women with higher BMI were less likely to smoke, consume alcohol, or be physically active, and more likely to have a lower socioeconomic status (Table 1). Women with a BMI of less than 20 kg/m^2 ^were more likely to smoke and to have a lower socioeconomic status, but less likely to drink alcohol or to be physically active, than women with a BMI of 20 to 24.9 kg/m^2^.

**Table 1:** Baseline characteristics and details of follow-up for coronary heart disease (CHD), by body mass index.

	Body mass index (kg/m^2^)					
		
	<20	20 to 24.9	25 to 29.9	30 to 34.9	≥35	All
	n = 44,787	n = 509,874	n = 420,712	n = 145,551	n = 58,015	n = 1,178,939
Baseline characteristics						
Age (years), mean (SD)	55.9 (5.0)	55.8 (4.8)	56.2 (4.8)	56.2 (4.8)	55.6 (4.6)	56.0 (4.8)
Current smoker,%	32.6	21.5	19.1	16.8	15.0	20.2
Lowest third of socioeconomic status,%	32.4	28.1	32.7	38.4	44.9	32.0
Non-drinker of alcoholic beverage,%	26.4	19.0	22.8	29.8	37.5	22.9
Rarely/never engage in strenuous physical activity,%	47.0	41.6	48.8	57.4	64.1	47.4
Follow-up (excluding first 4 years)						
Person-years for first coronary event (1,000s)	223	2,592	2,105	716	280	5,916
Person-years for CHD death (1,000s)	225	2,618	2,134	729	285	5,992
Women with first coronary event, n	999	11,005	12,498	5,363	2,600	32,465
Women with CHD as underlying cause of death, n	117	811	856	377	270	2,431

Percentages are calculated based on women with complete information for that specific variable.

After an average of 9 years of follow-up, there were 48,842 first coronary events including 5,097 CHD deaths. The annual incident CHD rates for the first 4 years of follow-up were slightly lower than the rates in the remaining follow-up period (see Additional file 1, Figure S1). To reflect the usual disease rates in the cohort, and to reduce the possibility of reverse causation (that preclinical disease might affect weight), we excluded the first 4 years of follow-up in all our subsequent analyses. In the remaining approximately 5 years of follow-up, there were 32,465 women with a first coronary event and 2,431 women who had CHD recorded as the underlying cause of death, corresponding to rates of 5.49 (95% CI 5.44 to 5.55) and 0.41 (95% CI 0.39 to 0.42) per 1,000 person-years, respectively.

**Figure 1:**
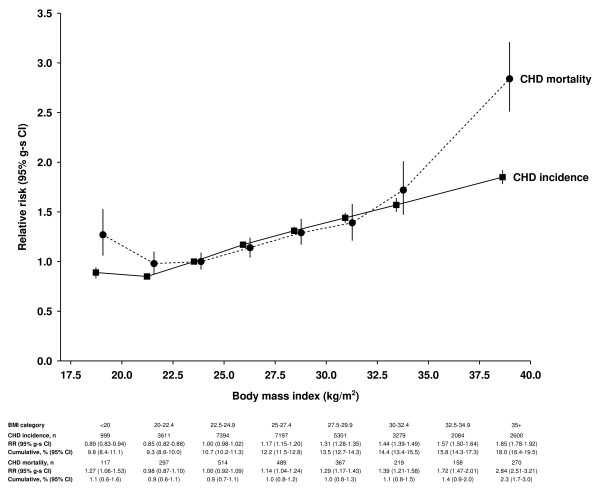
**Adjusted relative risk (95% group-specific confidence interva; g-sCI) for coronary heart disease (CHD) incidence and mortality in relation to body mass index (BMI)**. Relative risks (RRs) are plotted against mean BMI in the corresponding BMI category. RR = 1.0 for women with BMI of 22.5 to 24.9 kg/m^2^. CHD cumulative incidence and mortality over 20 years from the age of 55 years. Spaces are provided between incidence and mortality RRs within each BMI category to distinguish their corresponding CIs.

From the lowest BMI categories of less than 20 kg/m^2 ^and 20 to 22.4 kg/m^2^, the relative risk of incident CHD increased progressively with BMI (Figure 1). The relative risk for incident CHD per increment of 5 kg/m^2 ^in BMI was 1.29 (95% CI 1.28 to 1.30) after adjustment for age and stratification by region. Additionally adjusting for smoking habit, physical activity, alcohol consumption and socioeconomic class slightly attenuated the relative risk to 1.23 (95% CI 1.22 to 1.25). For CHD mortality, the relationship with BMI was J-shaped, and the pattern of risk differed from that of incident disease, with the relative risk being greater for CHD mortality than for incident outcomes among women in the lowest and highest categories of BMI. Compared with a BMI of 22.5 to 24.9 kg/m^2^, the relative risk of a BMI of less than 20 kg/m^2 ^for CHD mortality was significantly raised at 1.27 (95% CI 1.06 to 1.53), but for incident coronary event was significantly reduced at 0.89 (95% CI 0.83 to 0.94). For BMI ≥35 kg/m^2^, the relative risk of 2.84 (95% CI 2.51 to 3.21) for CHD mortality was considerably greater than the relative risk of 1.85 (95% CI 1.78 to 1.92) for incident disease.

**Figure 2:**
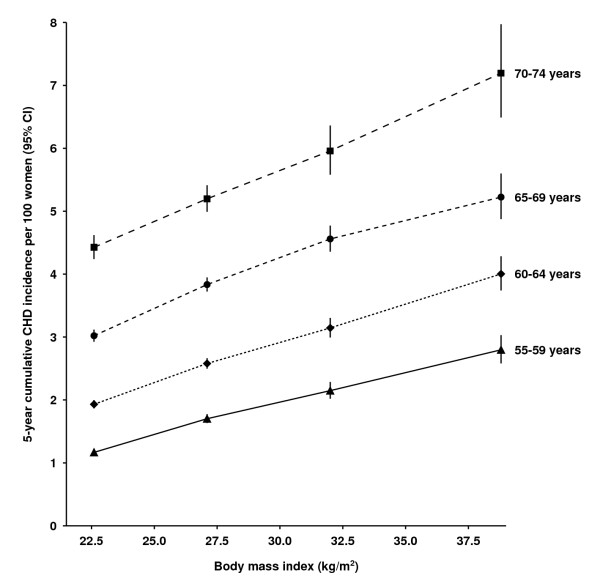
**Cumulative incidence (95% confidence interval; CI) of coronary heart disease (CHD) over 5 years in relation to body mass index (BMI) and attained age**. Cumulative incidences are plotted against mean BMI in the corresponding BMI category.

The incidence of CHD increased with age, and the progressive increase in risk with BMI was evident in each 5-year age group from 55 to 59 years to 70 to 74 years (Figure 2; see Additional file 1, Table S1 for further details). Moreover, a BMI increase of 10 kg/m^2 ^conferred an additional CHD risk similar to that conferred by a 5-year increase in age. The 20-year cumulative incidence of the disease was 12.1% (95% CI 11.9 to 12.2), that is, about one in eight women (12%) in this cohort had a first coronary event in the 20 years from age 55 to 74 years. Within the range of 20 to 34.9 kg/m^2 ^(*n *= 28,866 incident coronary events), there was a sufficient number of events to show that the incidence increased in a graded manner with small increments in BMI (see Additional file 1, Figure S2). The 20-year cumulative incidence of the disease for a BMI of 34 kg/m^2 ^was almost twice that for a BMI of 21 kg/m^2 ^(16.7% (95% CI 13.9 to 19.4%) versus 9.2% (95% CI 8.1 to 10.3%)). This means that around 1 in 6 (95% CI 1 in 5 to 7) women with a BMI of 34 kg/m^2^, compared with 1 in 11 (95% CI 1 in 10 to 12) women with a BMI of 21 kg/m^2^, had a first coronary event over a 20-year period from age 55 years.

**Figure 3:**
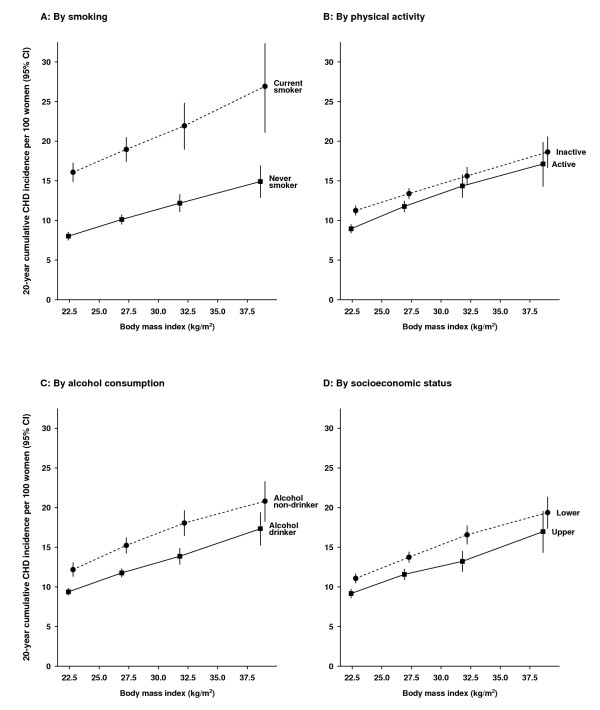
**The 20-year cumulative coronary heart disease (CHD) incidence (95% confidence interval; CI) from age 55 to 74 years in relation to body mass index (BMI) and other risk factors**. Cumulative incidences are plotted against mean BMI in the corresponding BMI category.

Assessing the relation between BMI and 20-year cumulative incidence of CHD from age 55 to 74 years by smoking, alcohol consumption, physical activity, and socioeconomic status, the increasing incidence of the disease associated with rising BMI levels was apparent in all subgroups (Figure 3; see Additional file 1, Table S2 for further details). Of all factors examined, current smoking had the greatest effect on CHD incidence. Current smokers who were neither overweight nor obese had similar CHD risks to severely obese never smokers: the cumulative incidence was 16.1% (95% CI 14.9 to 17.3%) for current smokers with BMI less than 25 kg/m^2 ^(mean BMI = 22.6 kg/m^2^) and 14.9% (95% CI 12.8 to 16.9%) for never smokers with BMI of 35 kg/m^2 ^or over (mean BMI = 38.7 kg/m^2^). The greatest cumulative risk of CHD from age 55 to 74 years was seen for current smokers with BMI of 35 kg/m^2 ^or over (26.9% (95% CI 21.1 to 32.4%)), suggesting that about 1 in every 4 such women (95% CI 1 in 3 to 5) had a first coronary event in the 20-year period from the age of 55 years. The effect of obesity was also apparent for alcohol drinkers and non-drinkers alike, with the greatest cumulative CHD risk found for non-drinkers with BMI of 35 kg/m^2 ^or over, and the lowest for drinkers with BMI of less than 25 kg/m^2^.

Correction for measurement error and changes in BMI over time had little effect on the relative risk estimates associated with increasing BMI (see Additional file 1, Table S3). The relative risk for incident CHD per 5-unit increase in BMI was 1.23 (95% CI 1.22 to 1.25) before and 1.24 (95% CI 1.23 to 1.26) after the correction was applied. Limiting the analysis to women with no missing value for any covariate had little effect on the relative risk (1.23 (95% CI 1.22 to 1.25)). When we accounted for competing causes of death, the 20-year cumulative risks were only slightly reduced (see Additional file 1, Figure S3).

## Discussion

In this large cohort of middle-aged UK women, about one in every eight women will have a first hospital admission for or have died from CHD in the 20 years from the age of 55 to 74 years. The cumulative incidence of CHD over 20 years increased progressively with BMI, from about one in eleven women with a BMI of 21 kg/m^2 ^to one in six women with a BMI of 34 kg/m^2^. Similar trends of progressively increasing CHD incidence with increasing BMI were seen across the age groups studied, and in smokers and non-smokers, alcohol drinkers and non-drinkers, women who were active and inactive, and in women from upper and lower socioeconomic groups.

Unlike the association between BMI and incident disease, there was a J-shaped relationship between BMI and CHD mortality. Our findings are consistent with findings from other large-scale prospective studies for CHD mortality [6-8,33] and for all-cause mortality (where a substantial proportion of deaths are due to vascular causes) [6,8,34-36]. It is possible that the relation with BMI varies for fatal and incident CHD outcomes [9-11], but these findings have been based on relatively small numbers of disease events. No other large-scale study has directly compared the BMI-CHD relationship for incident and fatal CHD. In our study, the relative risks for those with the lowest and highest BMI values were greater in magnitude for CHD mortality than for incident coronary event, suggesting that case fatality rates are higher in both lean and obese women than in those with a BMI within the normal or overweight range. The underlying reason for the excess vascular deaths associated with low BMI remains unclear. Although we excluded early disease events, reverse causation remains a possibility, as the effect of preclinical illness in those with low BMI on fatal outcomes may persist over a long period [36]. However, this confounding effect was not apparent for incident CHD events as we found no evidence of an increased incidence rate associated with low BMI. Thus, our findings provide evidence for the importance of greater adiposity in the occurrence of a CHD event. In other studies that investigated the association between BMI and incident CHD [9,12-15,17,37], results were broadly similar to those found here, but generally lacked power to describe reliably the relation between BMI and incident CHD across a wide range of values and important subgroups in the population.

BMI is known to vary with a number of factors, including age, smoking, physical activity, alcohol consumption, and socioeconomic status [8,15,38], which are also associated with CHD risk [2,3]. We took these potential confounding factors into account in our analyses. Further, our results suggest that obesity remains as important a risk factor for incident disease in older as in younger women in the age group studied. The effect of a 10 kg/m^2 ^increment in BMI on CHD incidence was comparable with that of a 5-year increase in chronological age. We also found that the increasing CHD risk with increasing BMI was consistently seen for current and never smokers, alcohol drinkers and non-drinkers, physically active and inactive women, and women in the upper and lower socioeconomic groups. Few studies have compared the combined effect of obesity and smoking on heart disease [15,18,19,37,39], and our results indicate a very large absolute risk that one in four current smokers who are also obese will develop a coronary event in the 20 years from the age of 55 years. Women drinkers in this cohort consume only low to moderate amounts of alcohol, and such consumption has been known to be associated with lower CHD risk [40]; nevertheless, CHD risk increased with BMI in both drinkers and non-drinkers.

Our findings could have important implications for public health, and suggest that reducing this obesity-associated CHD burden will probably require population-level preventive strategies. One such approach suggests shifting the population distribution of a modifiable risk factor downwards to prevent the occurrence of CHD [5]. However, shifting the population distribution of BMI downwards may have unintended consequences, considering that large-scale prospective studies that looked at fatal disease have shown increased CHD mortality risks with both high and low BMI levels [6-8]. However, our findings suggest that there is no such increased risk at the lower end of the BMI range for incident outcomes. Because most CHD events occurred in non-obese women, and small increments in BMI were associated with increasing incidence of the disease, small shifts in BMI distribution could potentially have a large effect on reducing the CHD burden both in the whole population and in important subgroups defined by age, smoking, physical activity level, alcohol consumption, and socioeconomic class.

There are some limitations to consider in interpreting our findings. We used self-reported weight and height at baseline to calculate BMI, and this can change over time. However, the correlation of measured and self-reported data for BMI is high, and correction for both measurement error and changes over time did not materially alter our results. We assessed only BMI, an indicator of total adiposity, but abdominal adiposity may also confer additional CHD risk [13,15]. Our findings included women only, but there is little to suggest that the relation between BMI and CHD differs between men and women [14,15,37]. However, the absolute risks may not be directly applicable to women in different populations. Allowing for competing causes of death hardly changed the 20-year incidence rates. Further, CHD may be clinically under-diagnosed in women [41]. However, mediating factors such as hypertension and dyslipidemia are known to increase with BMI [8,15] and so the proportion of undiagnosed cases may be higher at the higher than the lower end of the BMI distribution. Thus, our estimates of the relative difference in incidence rates between lower and higher BMI levels are likely to be conservative. Study participants were recruited when they were invited for routine breast cancer screening by a nationwide program. At the time the cohort was recruited, study participants represented one in four of the UK women in the target age range [20], so our findings are likely to be relevant to a large proportion of middle-aged women.

## Conclusions

The effect of obesity on CHD was substantial in this cohort of women, and the association was consistently seen in various subgroups defined by their age, smoking, physical activity, alcohol consumption, and socioeconomic class. Because most CHD events occurred in non-obese women, and even small increments in BMI were associated with increasing incidence of the disease, small shifts in the population distribution of BMI may potentially have a large effect on reducing the CHD burden in the population.

## Abbreviations

BMI: body mass index; CHD: coronary heart disease; CI: confidence interval; g-sCI: group-specific confidence interval; ICD-10: *International Statistical Classification of Diseases and Related Health Problems*: Tenth Revision; NHS: National Health Service.

## Competing interests

The authors declare that they have no competing interests.

## Authors' contributions

VB, GR, and JG were involved in the conception, design, and data acquisition for the Million Women Study. DC, BJC, AB, FLW, and VB analyzed and interpreted the data. DC drafted the first version of the manuscript. BJC, AB, FLW, JG, GR, and VB gave critical intellectual input and contributed to drafting revised versions of the manuscript. All authors gave their final approval of the version to be published.

## Pre-publication history

The pre-publication history for this paper can be accessed here:

http://www.biomedcentral.com/1741-7015/11/87/prepub

## Supplementary Material

Additional file 1 **Figure S1**. Body mass index and annual coronary heart disease (CHD) incidence (95% confidence interval) in relation to year of follow-up from baseline. **Table S1**. Cumulative incidence (95% confidence interval) of coronary heart disease in relation to body mass index and attained age (supplement for Figure 2). **Figure S2**. The 20-year cumulative incidence of coronary heart disease (CHD) from age 55 to 74 years in relation to body mass index. **Table S2**. Number of incident coronary heart disease (CHD) events in relation to body mass index and other risk factors (supplement for Figure 3). **Table S3**. Relative risk (95% confidence interval (CI)) of coronary heart disease per 5 kg/m^2 ^increase in body mass index and correction for measurement error by regression calibration. **Figure S3**. The 20-year cumulative incidence of coronary heart disease (CHD) (cause-specific and with competing causes of deaths) from age 55 to 74 years in relation to body mass index. Click here for file
