# Supplementary material for: Body mass index and incident coronary heart disease in women: a population-based prospective study
Source: BMC Med. 2013 Apr 2;11:87. doi: 10.1186/1741-7015-11-87 (PMC3661394; doi:10.1186/1741-7015-11-87)

Additional file 1

Online supplement to: D Canoy, B J Cairns, A Balkwill, F L Wright, J Green, G Reeves and V Beral. *Body mass index and incident coronary heart disease in women: a population-based prospective study*.

**Figure S1.** Body mass index and annual coronary heart disease (CHD) incidence (95% confidence interval) in relation to year of follow-up from baseline. **Page 2**

**Table S1.** Cumulative incidence (95% confidence interval) of coronary heart disease in relation to body mass index and attained age (supplement for Figure 2). **Page 3**

**Figure S2.** The 20-year cumulative incidence of coronary heart disease (CHD) from age 55 to 74 years in relation to body mass index. **Page 4**

**Table S2.** Number of incident coronary heart disease (CHD) events in relation to body mass index and other risk factors (supplement for Figure 3). **Page 5**

**Table S3.** Relative risk (95% confidence interval (CI)) of coronary heart disease (CHD) per 5 kg/m^2^ increase in body mass index and correction for measurement error by regression calibration. **Page 6**

**Figure S3.**The 20-year cumulative incidence of coronary heart disease (CHD) (cause-specific and with competing causes of deaths) from age 55 to 74 years in relation to body mass index.  **Page 7**

Figure S1. Body mass index and annual coronary heart disease (CHD) incidence (95% confidence interval [CI]) in relation to years of follow-up from baseline. Rates are stratified by region and uniformly age-standardised to match the average CHD rate for each year between ages 50 to 74 years.


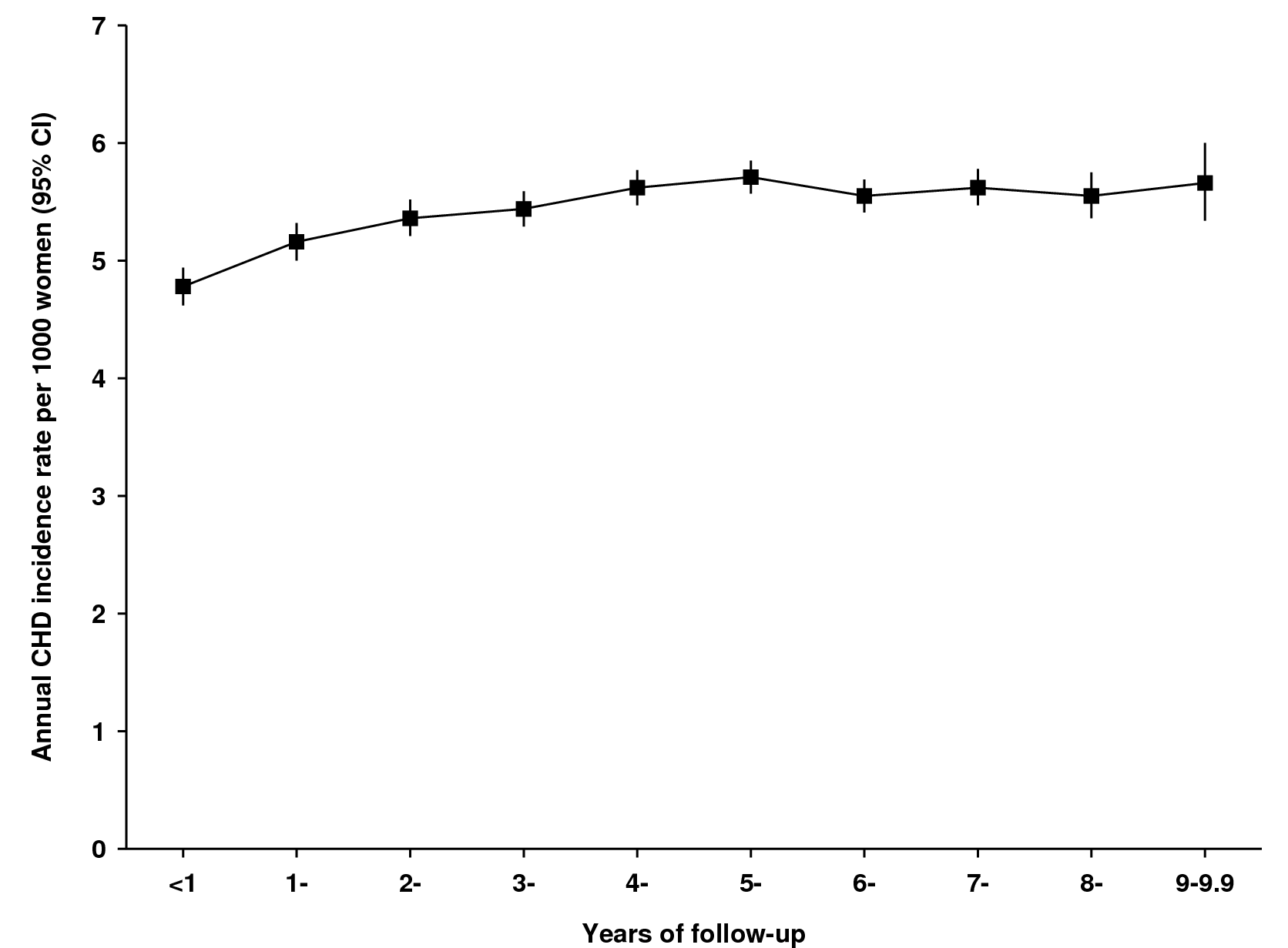


|  | Follow-up (years) | | | | | | | | | |
| --- | --- | --- | --- | --- | --- | --- | --- | --- | --- | --- |
|  | <1 | 1 to 1.9 | 2 to 2.9 | 3 to 3.9 | 4 to 4.9 | 5 to 5.9 | 6 to 6.9 | 7 to 7.9 | 8 to 8.9 | 9 to 9.9 |
| No. with CHD | 2,781 | 3,628 | 4,228 | 4,750 | 5,167 | 5,719 | 6,209 | 6,295 | 5,495 | 3,374 |
| Person-years | 1,177 | 1,171 | 1,164 | 1,156 | 1,146 | 1,135 | 1,121 | 1,082 | 879 | 517 |
| Incident rate per 1,000 (95% CI) | **4.78** (4.62 to 4.94) | **5.16** (5.00 to 5.32) | **5.36** (5.21 to 5.52) | **5.44** (5.29 to 5.59) | **5.62** (5.47 to 5.77) | **5.71** (5.57 to 5.85) | **5.55** (5.41 to 5.69) | **5.62** (5.47 to 5.78) | **5.55** (5.36 to 5.75) | **5.66** (5.34 to 6.00) |

Table S1. Cumulative incidence (95% confidence interval (CI)) of coronary heart disease (CHD) in relation to body mass index and attained age (supplement for Figure 2).

|  | **Body mass index (kg/m^2^)** | | | |  |
| --- | --- | --- | --- | --- | --- |
|  | **<25** | **25 to 29.9** | **30 to 34.9** | **≥35** | **All** |
| **5-year cumulative incidence** |  |  |  |  |  |
| From age 55 to 59 years |  |  |  |  |  |
| No. of women (No. with CHD) | 54,951 (2,036) | 38,078 (2 131) | 13,859 (1,001) | 6,707 (603) | 113,595 (5,771) |
| Cumulative incidence per 100 women (95% CI) | 1.2 (1.1 to 1.2) | 1.7 (1.6 to 1.8) | 2.1 (2.0 to 2.3) | 2.8 (2.6 to 3.0) | 1.5 (1.5 to 1.6) |
| From age 60 to 64 years |  |  |  |  |  |
| No. of women (No. with CHD) | 217,026 (3,415) | 152,598 (3,534) | 53,169 (1,542) | 22,439 (825) | 445,232 (9,316) |
| Cumulative incidence per 100 women (95% CI) | 1.9 (1.9 to 2.0) | 2.6 (2.5 to 2.7) | 3.1 (3.0 to 3.3) | 4.0 (3.7 to 4.3) | 2.4 (2.4 to 2.5) |
| From age 65 to 69 years |  |  |  |  |  |
| No. of women (No. with CHD) | 144,407 (3,914) | 114,194 (4,245) | 38,553 (1,807) | 14,623 (777) | 311,777 (10,743) |
| Cumulative incidence per 100 women (95% CI) | 3.0 (2.9 to 3.1) | 3.8 (3.7 to 3.9) | 4.6 (4.4 to 4.8) | 5.2 (4.9 to 5.6) | 3.7 (3.6 to 3.8) |
| From age 70 to 74 years |  |  |  |  |  |
| No. of women (No. with CHD) | 96,927 (2,122) | 83,039 (2,153) | 28,278 (859) | 9,572 (344) | 217,816 (5,478) |
| Cumulative incidence per 100 women (95% CI) | 4.4 (4.2 to 4.6) | 5.2 (5.0 to 5.4) | 6.0 (5.6 to 6.4) | 7.2 (6.5 to 8.0) | 5.0 (4.9 to 5.3) |
| **20-year cumulative incidence** |  |  |  |  |  |
| From age 55 to 74 years |  |  |  |  |  |
| No. of women (No. with CHD) | 513,311 (11,487) | 387,909 (12,063) | 133,859 (5,209) | 53,341 (2,549) | 1,088,420 (31,308) |
| Cumulative incidence per 100 women (95% CI) | 10.2 (9.7 to 10.6) | 12.7 (12.2 to 13.2) | 14.9 (14.1 to 15.8) | 17.9 (16.3 to 19.5) | 12.1 (11.9 to 12.2) |

Figure S2.The 20-year cumulative incidence of coronary heart disease (CHD) from age 55 to 74 years in relation to body mass index.


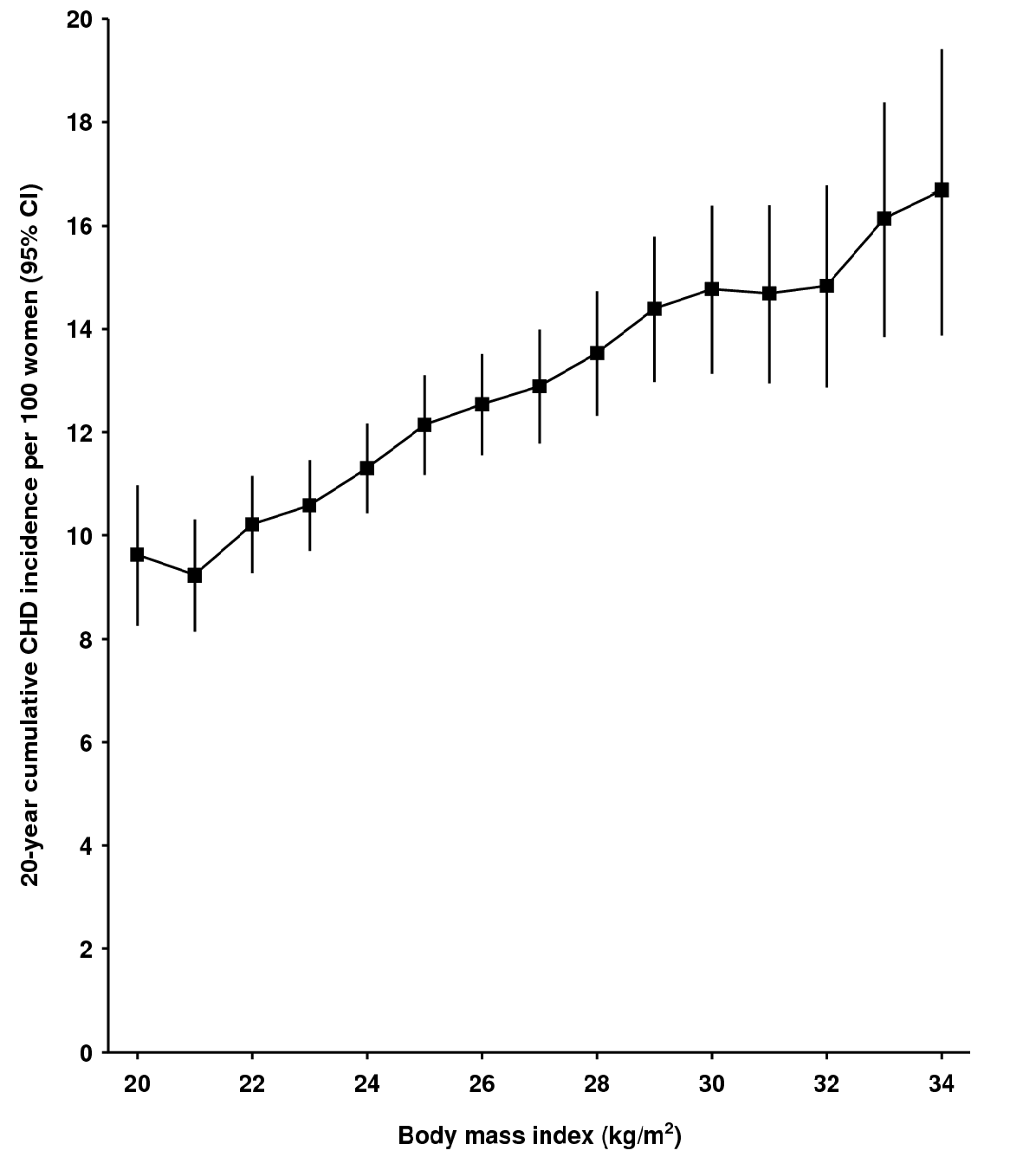


| **Body mass index, kg/m^2^** | **No. of women** | **No. with CHD** | **20-year cumulative incidence**  **per 100 (95% CI) from age 55 years** |
| --- | --- | --- | --- |
| 20 to 20.9 | 48,685 | 1,030 | 9.6 (8.3 to 11.0) |
| 21 to 21.9 | 77,286 | 1,507 | 9.2 (8.1 to 10.3) |
| 22 to 22.9 | 109,879 | 2,380 | 10.2 (9.3 to 11.1) |
| 23 to 23.9 | 123,695 | 2,848 | 10.6 (9.7 to 11.5) |
| 24 to 24.9 | 126,437 | 3,240 | 11.3 (10.4 to 12.2) |
| 25 to 25.9 | 103,633 | 2,947 | 12.1 (11.2 to 13.1) |
| 26 to 26.9 | 98,099 | 2,967 | 12.5 (11.6 to 13.5) |
| 27 to 27.9 | 78,817 | 2,468 | 12.9 (11.8 to 14.0) |
| 28 to 28.9 | 67,609 | 2,261 | 13.5 (12.3 to 14.7) |
| 29 to 29.9 | 51,053 | 1,855 | 14.4 (13.0 to 15.8) |
| 30 to 30.9 | 39,654 | 1,466 | 14.8 (13.1 to 16.4) |
| 31 to 31.9 | 33,532 | 1,285 | 14.7 (12.9 to 16.4) |
| 32 to 32.9 | 26,023 | 1,034 | 14.8 (12.9 to 16.8) |
| 33 to 33.9 | 22,123 | 912 | 16.1 (13.8 to 18.4) |
| 34 to 34.9 | 15,858 | 666 | 16.7 (13.9 to 19.4) |

Table S2. Number of incident coronary heart disease (CHD) events in relation to body mass index and other risk factors (supplement for Figure 3).

| **Body mass index, kg/m^2^** | **Risk factors** | | | |
| --- | --- | --- | --- | --- |
|  | % | No. with CHD / N of women | % | No. with CHD / N of women |
| **A. By smoking (N=1,170,462)*†** |  |  |  |  |
|  |  | Never smokers |  | Current smokers |
| <25 | 1.7 | 4,260 / 254,459 | 3.7 | 4,027 / 108,121 |
| 25 to 29.9 | 2.4 | 4,601 / 189,405 | 4.8 | 3,316 / 69,180 |
| 30 to 34.9 | 3.2 | 2,057 / 65,176 | 5.8 | 1,211 / 20,877 |
| ≥35 | 4.0 | 1,016 / 25,658 | 7.3 | 538 / 7,332 |
| **B. By physical activity (N=1,139,759)*** | |  |  |  |
|  |  | Active |  | Inactive |
| <25 | 1.8 | 5,129 / 291,455 | 2.9 | 5,877 / 206,150 |
| 25 to 29.9 | 2.6 | 5,022 / 194,122 | 3.6 | 6,495 / 180,995 |
| 30 to 34.9 | 3.3 | 1,817 / 55,722 | 4.3 | 3,155 / 73,308 |
| ≥35 | 4.1 | 770 / 18,696 | 5.1 | 1,650 / 32,639 |
| **C. By alcohol consumption (N=1,171,446)*** | |  |  |  |
|  |  | Drinker |  | Non-drinker |
| <25 | 2.0 | 8,118 / 412,074 | 3.3 | 3,253 / 98,427 |
| 25 to 29.9 | 2.7 | 8,191 / 298,875 | 4.3 | 3,740 / 86,584 |
| 30 to 34.9 | 3.3 | 3,124 / 93,756 | 5.2 | 2,018 / 39,091 |
| ≥35 | 4.1 | 1,354 / 33,296 | 5.9 | 1,155 / 19,580 |
| **D. By socioeconomic status (N=1,270,314)*** | |  |  |  |
|  |  | Upper half |  | Lower half |
| <25 | 1.8 | 5,005 / 273,818 | 2.7 | 6,396 / 235,697 |
| 25 to 29.9 | 2.6 | 4,876 / 189,632 | 3.6 | 7,090 / 195,464 |
| 30 to 34.9 | 3.1 | 1,788 / 57,652 | 4.5 | 3,388 / 75,209 |
| ≥35 | 4.0 | 790 / 19,945 | 5.3 | 1,745 / 33,026 |

*N of women with complete values for the variable; †Includes former smokers.

Table S3. Relative risk (95% confidence interval (CI)) of coronary heart disease (CHD) per 5 kg/m^2^ increase in body mass index and correction for measurement error by regression calibration.

| **Outcome** | **No. of women**  **(No. with CHD)** | **Regression dilution ratio (95% CI)** | **Relative risk (95% CI)** | |
| --- | --- | --- | --- | --- |
|  |  |  | **Uncalibrated** | **Calibrated** |
| Incident CHD | 1,118,722 (32,465) | 0.97 (0.94 to 0.99) | 1.23 (1.22 to 1.25) | 1.24 (1.23 to 1.26) |
| CHD mortality | 1,118,722 (2,431) | 0.97 (0.94 to 0.99) | 1.32 (1.28 to 1.36) | 1.33 (1.29 to 1.38) |

**Figure S3.**The 20-year cumulative incidence of coronary heart disease (CHD) (cause-specific and with competing causes of deaths) from age 55 to 74 years in relation to body mass index.


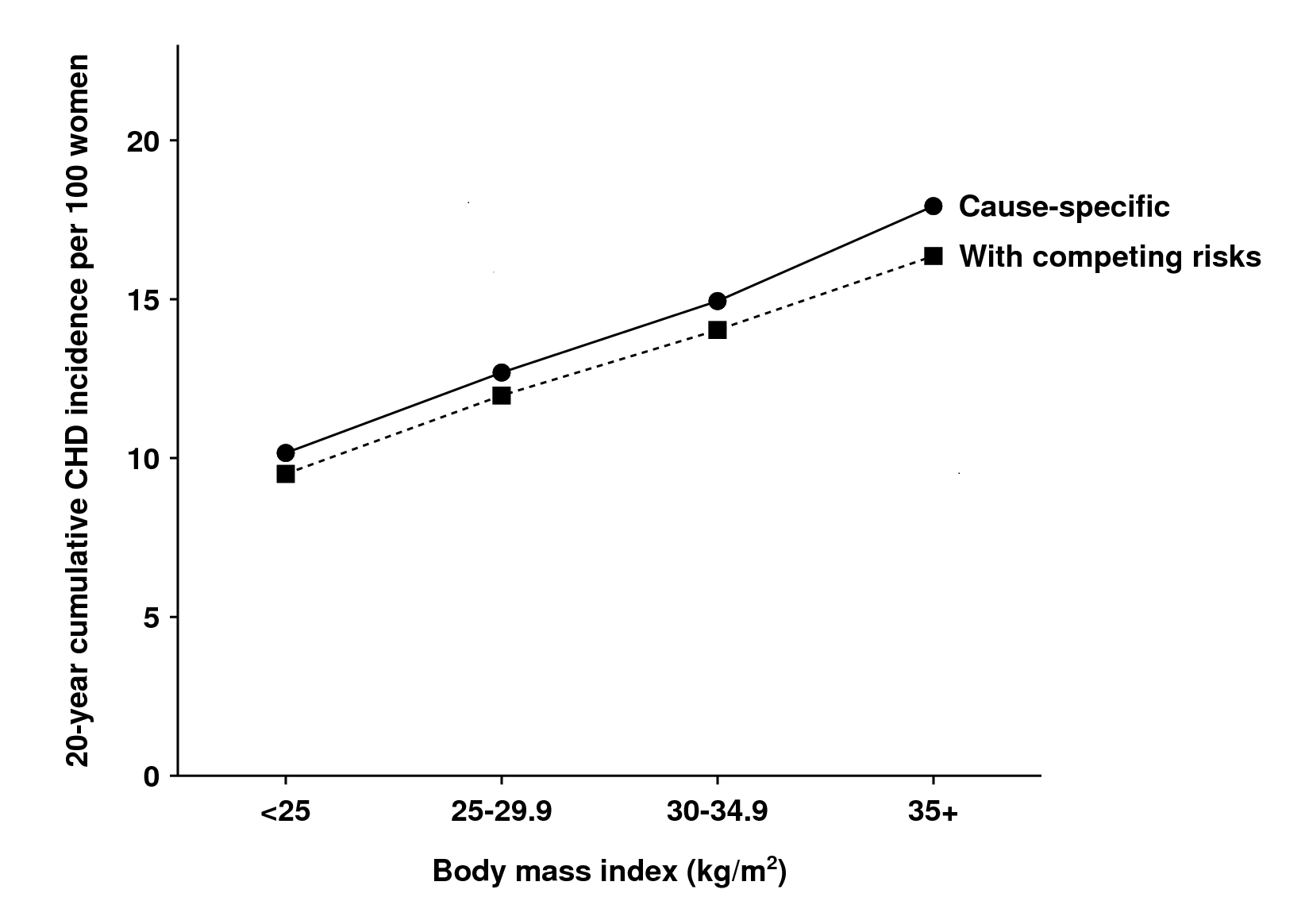

Supplement: Additional file 1 — Figure S1. Body mass index and annual coronary heart disease (CHD) incidence (95% confidence interval) in relation to year of follow-up from baseline. Table S1. Cumulative incidence (95% confidence interval) of coronary heart disease in relation to body mass index and attained age (supplement for Figure 2). Figure S2. The 20-year cumulative incidence of coronary heart disease (CHD) from age 55 to 74 years in relation to body mass index. Table S2. Number of incident coronary heart disease (CHD) events in relation to body mass index and other risk factors (supplement for Figure 3). Table S3. Relative risk (95% confidence interval (CI)) of coronary heart disease per 5 kg/m2 increase in body mass index and correction for measurement error by regression calibration. Figure S3. The 20-year cumulative incidence of coronary heart disease (CHD) (cause-specific and with competing causes of deaths) from age 55 to 74 years in relation to body mass index. [file 1741-7015-11-87-S1.DOCX]
